# Scalable, cell type-selective, AAV-based *in vivo* CRISPR screening in the mouse brain

**DOI:** 10.1101/2023.06.13.544831

**Published:** 2023-06-27

**Authors:** Biswarathan Ramani, Indigo V.L. Rose, Andrew Pan, Ruilin Tian, Keran Ma, Jorge J. Palop, Martin Kampmann

**Affiliations:** 1. Institute for Neurodegenerative Diseases, University of California, San Francisco, San Francisco, CA, USA; 2. Department of Pathology, University of California, San Francisco, San Francisco, CA, USA; 3. Neuroscience Graduate Program, University of California, San Francisco, San Francisco, CA, USA; 4. Biophysics Graduate Program, University of California, San Francisco, San Francisco, CA, USA; 5. Gladstone Institute of Neurological Disease, San Francisco, CA, USA; 6. Department of Neurology, University of California, San Francisco, San Francisco, CA, USA; 7. Weill Institute for Neurosciences, University of California, San Francisco, San Francisco, CA, USA; 8. Department of Biochemistry and Biophysics, University of California, San Francisco, San Francisco, CA, USA

## Abstract

CRISPR-based genetic screening directly in mammalian tissues *in vivo* is challenging due to the need for scalable, cell-type selective delivery and recovery of guide RNA libraries. We developed an *in vivo* adeno-associated virus-based and Cre recombinase-dependent workflow for cell type-selective CRISPR interference screening in mouse tissues. We demonstrate the power of this approach by identifying neuron-essential genes in the mouse brain using a library targeting over 2000 genes.

## Intro

CRISPR-based genetic screens are powerful tools for biological discovery since they enable the massively parallel interrogation of gene function. The majority of such screens has been carried out in cultured cells, where first applications in iPSC-derived brain cell types such as neurons^1,2^, microglia^3^, and astrocytes^4^ have uncovered cell type-specific mechanisms relevant to neuroscience and neurological diseases.

However, CRISPR-based screens in cultured mammalian cells do not recapitulate the physiological context of a live, multicellular organism, or of tissue states such as aging, inflammation, or disease. These limitations are particularly obvious in the case of complex organs such as the brain, which involves intricate spatial interactions between a large number of distinct cell types and subtypes. Therefore, the implementation of *in vivo* pooled CRISPR screens in the brains of animals such as mice has the potential to uncover insights into brain function and disease that would be elusive in cell culture.

**Extended Data Fig. 1:**
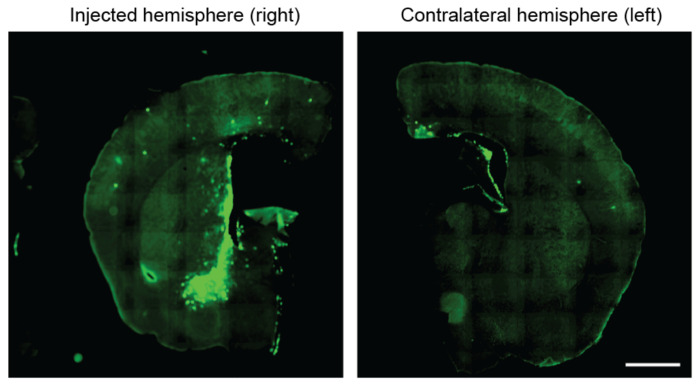
Distribution of lentivirus containing sgRNA in the mouse brain. Lentivirus containing pLG15 (Ref. ^17^) with a non-targeting sgRNA and a mTagBFP2 marker was injected into the neonate mouse by ICV, and brains were extracted on day 14 and sectioned coronally. The slices were stained using an anti-mTagFP antibody (signal shown in green). Sections were from the same brain. Scale bar 1 mm.

**Fig. 1:**
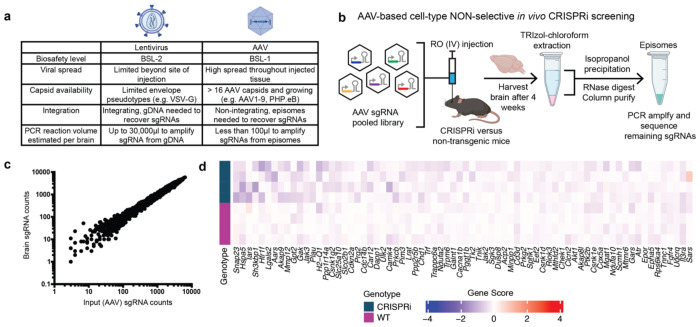
Pilot AAV-based pooled CRISPRi screen in mouse brains reveals challenges **a**, Comparison of lentiviral versus AAV-based delivery of sgRNA libraries to mouse tissues for direct *in vivo* CRISPR-based screening. **b**, Workflow for pooled non-cell type-selective screening using AAV delivery of an sgRNA library to the brain of CRISPRi or control non-transgenic wild-type (WT) mice. **c**, Frequencies of sgRNAs in the input AAV library compared to those recovered from a representative mouse brain. **d**, Results of the screen showing the genes with the most negative average Gene Score to identify neuron-essential genes for four CRISPRi and four WT mice (rows), FDR < 0.1. Very few consistent hit genes were identified.

A small number of *in vivo* CRISPR screens in mouse brains has previously been reported^5–8^. All but one of these screens were implemented by delivering sgRNA libraries to the brain via lentivirus, which has a number of drawbacks (Fig. 1a). First, available lentiviral envelope pseudotypes offer little control on tropism of different tissues and cell types. Hence, in complex tissues with many distinct cell types, such as the brain, lentiviral screens in which phenotypes such as survival are averaged over many cells cannot differentiate between genes affecting the phenotypes in different cell types^5^. Second, lentivirus injected into a tissue has limited spread beyond the region of injection (Extended Data Fig. 1), therefore limiting the scope and scale of screens. Third, recovery of sgRNA information requires PCR amplification of sgRNA-encoding regions from genomic DNA, which becomes very costly when applied to whole tissues, limiting scalability.

To address these challenges, we turned to adeno-associated virus (AAV) as a robust, versatile way to deliver sgRNAs to different tissues, with several advantages over lentivirus for *in vivo* screening applications (Fig. 1a). AAV spreads broadly from the site of injection, and a growing number of AAV capsids have been optimized for targeting different cell types, along with options for intravenous delivery^9^. Unlike lentivirus-based pooled CRISPR screening approaches, which require amplification of sgRNAs from genomic DNA for screen analysis, we reasoned that AAV-based approaches could enable amplification of sgRNA sequences on isolated AAV episomes, vastly reducing the cost of analysis (Fig. 1a).

Despite these advantages, only one AAV-based direct *in vivo* CRISPR screen in mouse brain has been reported^8^, which delivered an sgRNA library targeting tumor suppressor genes to trigger tumorigenesis and examined the edited genomic DNA of the resulting tumors, but did not recover sgRNAs from targeted cells. Here, we conducted a pilot screen using sgRNA amplifications from isolated episomes and detected an unexpected challenge likely due to the recovery of episomes from virions that had not entered the desired cell type. We describe a strategy for recovery of sgRNAs only from cells expressing Cre recombinase, enabling cell type-specific screens. We demonstrate the performance of this approach in a CRISPRi screen for neuron-essential genes.

## Results

### Non-cell type-specific, AAV-based CRISPRi pilot screen in mouse brains reveals challenges

We conducted a pilot *in vivo* screen using a pooled sgRNA library delivered via AAV (Fig. 1b) to test whether amplification of sgRNAs from AAV episomes isolated from whole brain would be a viable strategy for screen analysis. We generated an AAV backbone (pAP210) and inserted our previously established 12,350-element sgRNA library targeting 2,269 genes, including kinases and other druggable targets^10^. We packaged this AAV using the PHP.eB capsid, which can be delivered by intravenous injection and has strong CNS and neuronal tropism but can also transduce astrocytes and oligodendrocytes^11^. We intravenously delivered the pooled AAV sgRNA library into 9-week-old mice (n=4) that constitutively express CRISPR interference (CRISPRi) machinery (JAX strain #030000, see Methods) as well as into age-matched wild-type mice. After 4 weeks, we performed a TRIzol-chloroform extraction on whole brains, following a protocol previously used to capture AAV episomes for sequencing^12^, and PCR-amplified sgRNA-encoding cassettes to generate samples for next-generation sequencing (see Methods).

**Extended Data Fig. 2:**
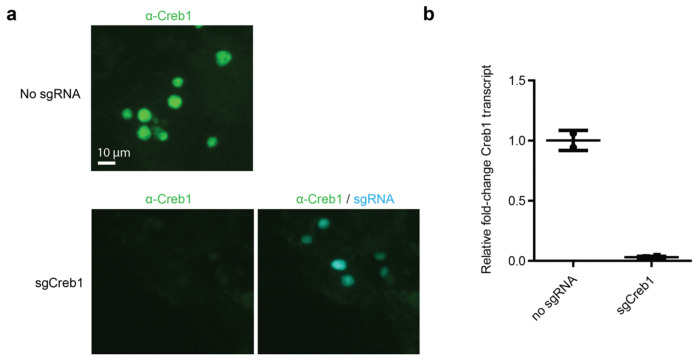
CRISPRi activity in cultured primary neurons of dCas9-KRAB mice. sgRNA targeting *Creb1* delivered by AAV into dCas9-KRAB primary neurons demonstrates marked knockdown of endogenous Creb1 by immunofluorescence (a) and by qRT-PCR (b, mean ± s.d., n = 2 technical replicates).

We confirmed full recovery of the sgRNA library from the brain with excellent correlation of sgRNA frequencies (Fig. 1c, Supplementary Table 1). However, the identified hits, knockdown of which decreases neuronal survival, only had a modest effect size and poor reproducibility between individual mice (Fig. 1d, Supplementary Tables 2). This lack of robust hits was likely not due to insufficient gene knockdown in the mice, since we confirmed strong CRISPRi activity in cultured primary neurons isolated from this mouse line (Extended Data Fig. 2). Instead, we hypothesized that episomes were recovered from a variety of cell types, thus diluting cell type-specific phenotypes, and that a substantial fraction of episomes might not have entered cells, but instead originate from non-transduced virions remaining in intravascular or interstitial spaces.

### Selective recovery of sgRNAs from Cre-expressing cells in the brain

**Fig. 2:**
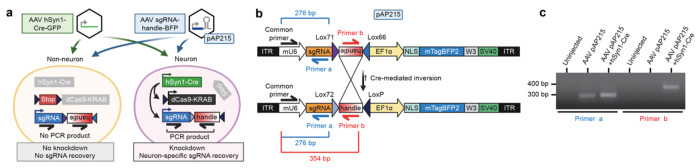
AAV backbone for cell type-specific, Cre-dependent sgRNA recovery **a**, Strategy for cell-type selective screening. **b**, The pAP215 AAV sgRNA backbone contains a lox71/lox66 flanked “handle” that undergoes inversion only in cells expressing Cre recombinase, allowing for cell type-specific sgRNA recovery by PCR. **c**, PCR performed on episomes recovered from mouse brains expressing AAV pAP215 with or without hSyn1-Cre-NLS-GFP (Cre) using the primers diagrammed in (b). PCR amplicons from brains of mice injected by ICV at P0 with pAP215 packaged into AAV PHP.eB, with or without co-injection of hSyn1-Cre, also packaged into AAV PHP.eB. The first three lanes represent samples from uninjected brain, pAP215 alone, and pAP215 + hSyn1-Cre with amplicons generated with Primer A, which captures presence of AAV episomes, but is not inversion specific. The next three lanes represent amplicons from the same samples generated with Primer B, which binds to the lox-flanked recombination site (handle) and generates amplicons only after Cre-mediated inversion.

To overcome this limitation, we developed a new AAV backbone (pAP215) for delivery of sgRNAs that enables Cre recombinase-dependent cell type-selective sgRNA recovery (Fig. 2a). In addition to expression modules for the sgRNA and a fluorescent marker (nuclear localized mTagBFP2), this plasmid backbone contains a “handle” sequence flanked by lox66 and lox71 sequences that undergo predominantly unidirectional inversion upon Cre recombinase expression^12,13^ (Fig. 2b). When used together with a promotor that drives Cre expression in a target cell type, sgRNAs from that cell type can be specifically recovered by PCR with an amplicon that spans the inverted handle sequence.

**Extended Data Fig. 3:**
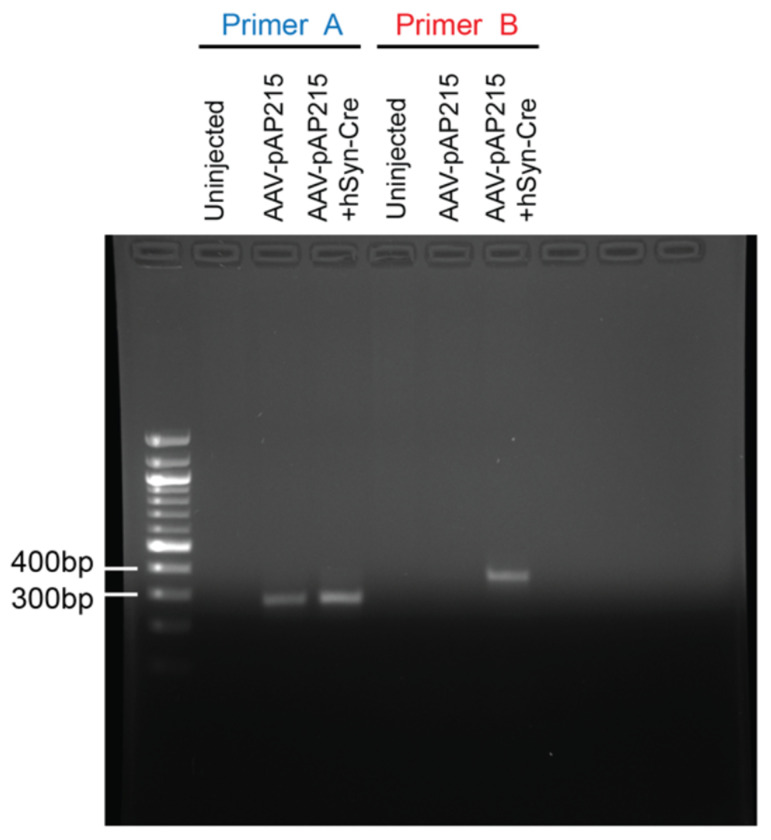
Full blot for figure 2.

To test this, we packaged pAP215 containing the sgRNA library into AAV using the PHP.eB capsid. We delivered this AAV pool, along with PHP.eB-packaged Cre recombinase expressed under the neuron-specific hSyn1 promoter (AAV.PHP.eB::hSyn1-Cre-NLS-GFP, hereafter referred to as hSyn1-Cre), by intracerebroventricular (ICV) injection into neonatal mice and harvested whole brains after three weeks to recover AAV episomes. We detected evidence of the inverted sequence only in mice co-injected with the Cre recombinase construct (Fig. 2c, Extended Data Fig. 3), confirming selective amplification of AAV episomes originating from Cre-expressing cells.

### Robust cell-type specific gene knockdown throughout the brain

**Extended Data Fig. 4:**
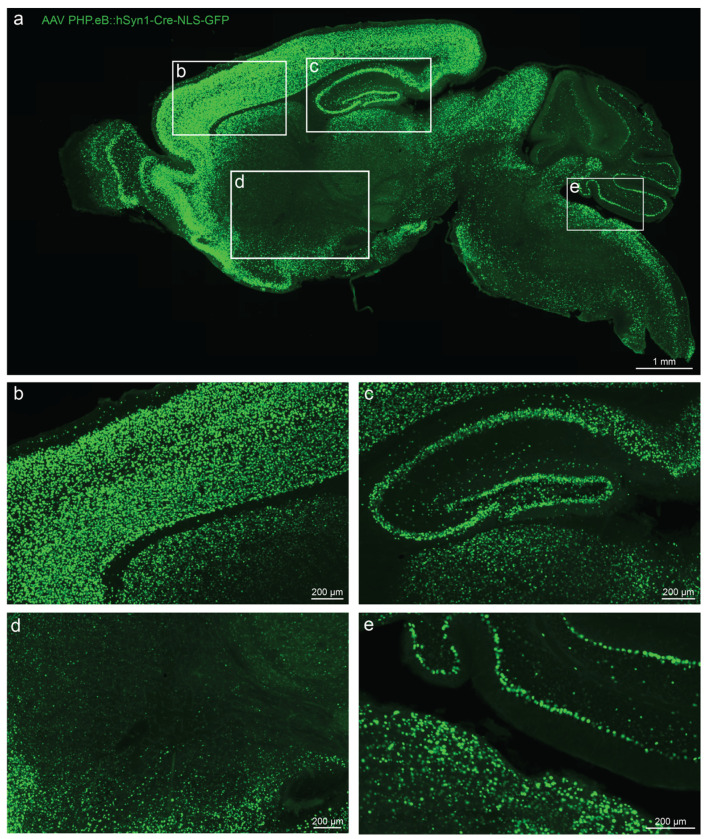
Distribution and expression of AAV in mouse brain. Sagittal section of mouse brain 3 weeks following ICV injection of AAV PHPe.B::hSyn1-Cre-NLS-GFP across the mouse brain (a), including cortex (b), hippocampus (c), striatum (d), and cerebellum (e). The same viral capsid, PHP.eB (ref ^18^), was used for delivery of all AAVs in this study. Image intensity is consistent across panels (a), (c), (d), and (e), but is reduced in panel (b) so individual neuronal nuclei are more easily resolved.

**Fig. 3:**
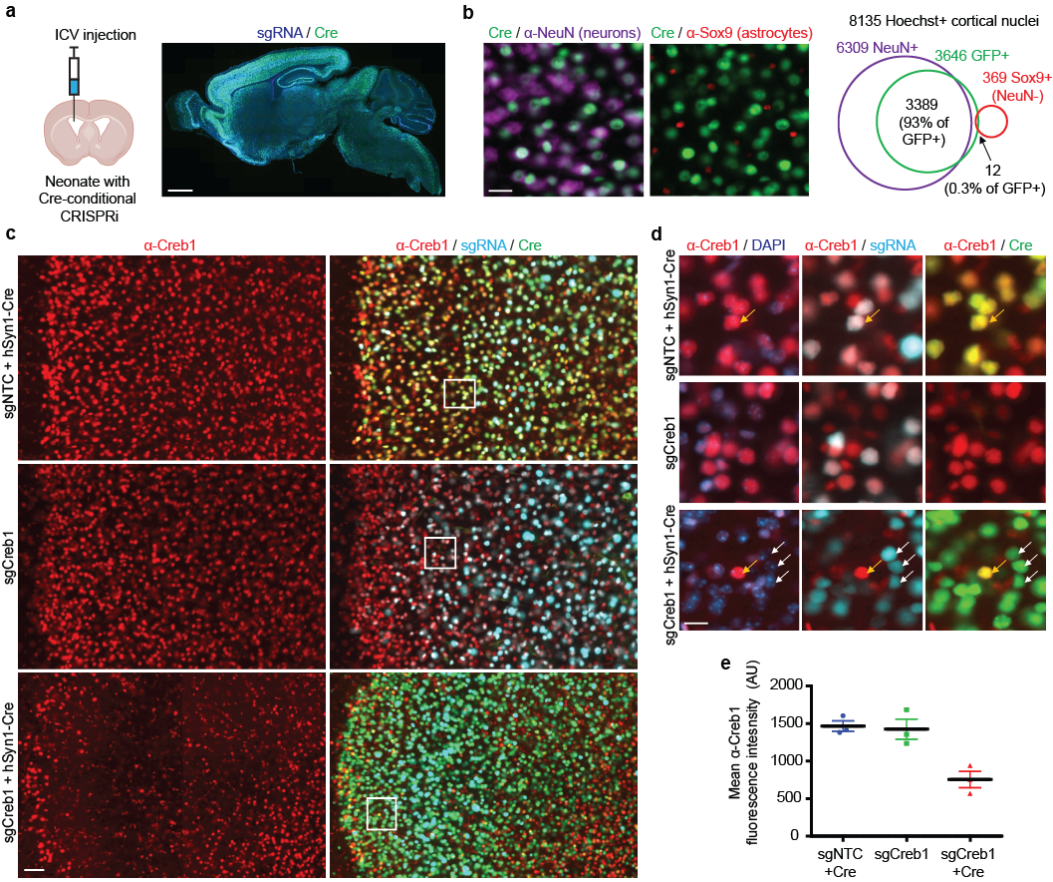
Cre-dependent CRISPRi knockdown of endogenous genes in neurons throughout the mouse brain **a**, Expression of AAV-pAP215 (sgRNA, based on BFP marker) and AAV-hSyn1-Cre-NLS-GFP (Cre, based on GFP) in a mouse brain three weeks after neonatal intracerebroventricular (ICV) delivery. Scale bar, 1 mm. **b**, Immunofluorescent staining for neurons (marker: NeuN) and astrocytes (marker: Sox9) in mouse cortex expressing AAV-hSyn1-Cre-NLS-GFP. Scale bar, 25 μm. Overlap of Cre expression with NeuN and Sox9 within a cortical region demonstrates high specificity for neurons and high coverage of neurons. **c**, sgRNAs targeting *Creb1* (sgCreb1) or a non-targeting control (sgNTC) in AAV-pAP215 were delivered with or without AAV hSyn1-Cre-NLS-GFP via ICV injection into neonatal mice with lox-stop-lox CRISPRi machinery. Brains were stained at 3 weeks with a representative area of frontal cortex shown. Scale bar, 50 μm. **d**, Higher magnification of the boxed regions in the left panels, with white arrows indicating examples of neurons having received both sgCreb1 and Cre, whereas yellow arrows indicate neurons that received Cre without sgCreb1. Scale bar, 10 μm. **e**, Quantification of Creb1 levels in sgRNA-positive nuclei within a representative region of cortex (mean ± s.e.m, n = 3 independent mice).

Next, we tested CRISPRi-mediated knockdown of genes by sgRNAs delivered via pAP215 into mice with Cre-dependent conditional CRISPRi machinery^14^. We delivered AAV by intracerebroventricular (ICV) injection into neonates. Histologic examination of brains transduced with the sgRNA-containing pAP215 (BFP+) and hSyn1-driven Cre (GFP+) confirmed widespread distribution of these AAVs throughout the mouse brain (Fig. 3a, Extended Data Fig. 4) and widespread and highly specific expression of hSyn1-Cre-GFP in neurons (Fig. 3b, Supplementary Table 3).

**Extended Data Fig. 5:**
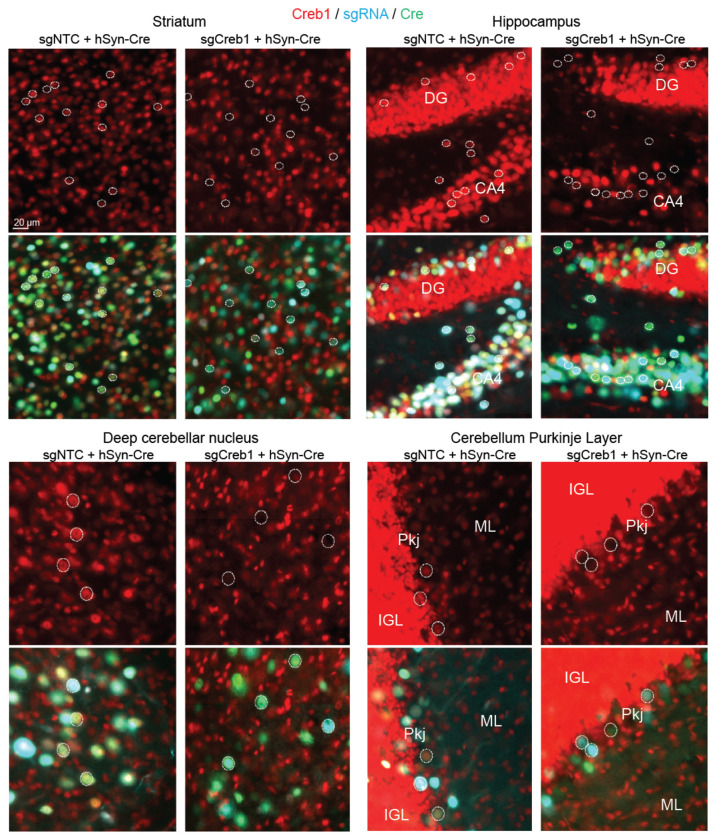
Cre-dependent CRISPRi knockdown of Creb1 in different mouse brain regions. Immunofluorescent staining for Creb1 in mice injected with AAV-packaged sgNTC or sgCreb1 (blue) along with hSyn1-Cre-NLS-GFP (green), for the indicated brain regions. Representative nuclei containing both BFP and GFP are outlined with a dotted circle, with the same nuclei shown in the top and bottom panels of each brain region, highlighting markedly reduced Creb1 in double-positive nuclei with sgCreb1 as compared to sgNTC. DG: dentate gyrus; ML: molecular layer, Pkj: Purkinje cell layer (Pkj); IGL: internal granule layer.

To evaluate the efficacy of CRISPRi-mediated gene knockdown in this system, we delivered an sgRNA targeting *Creb1*, a nuclear protein not essential for survival, or a non-targeting control sgRNA. Three weeks later, brain tissue showed robust Creb1 knockdown only in neurons transduced with both sgRNA and hSyn1-Cre AAVs (Fig. 3c-e, Extended Data Fig. 5).

### Cre-dependent CRISPRi screens identify neuron-essential genes

We inserted the 12,350-element sgRNA library described above into the pAP215 AAV backbone and delivered it along with hSyn1-Cre by ICV injection into conditional CRISPRi neonate mice.

**Fig. 4:**
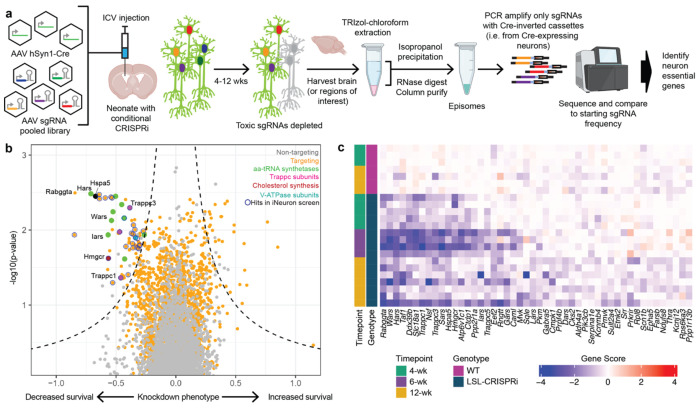
*In vivo* CRISPRi screen for neuron-essential genes in mouse brains **a**, Strategy for *in vivo* CRISPRi screening to identify neuron-essential genes. **b**, Knockdown phenotypes for 2,269 genes averaged across four mice at 12 weeks after ICV injection of an sgRNA library. Genes within enriched pathways are color-coded, and hits that overlap with essential genes in iPSC-derived neurons^1^ are circled in blue. Dashed lines indicate the 0.1 FDR cutoff. **c**, Phenotypes of neuron-essential genes from (b) with an FDR < 0.1 and negative Gene Score in individual mice (rows), and compared to conditional CRISPRi and wild-type mice at different time points.

We collected brains after 12 weeks, recovered episomes and amplified sgRNAs using primers specific to the Cre-inverted handle in pAP215, followed by next-generation sequencing (Fig. 4a). Each screen replicate was accomplished in an individual mouse without pooling.

Analysis of changes in sgRNA frequencies identified several strong hits, knockdown of which decreased neuronal survival (Fig. 4b, Supplementary Table 3). Several genes belonged to distinct biological categories, including tRNA synthetases (*Wars, Hars, Sars*) or components of the endolysosomal pathway (*Atp6v1c1, Rabggta*). Many of the neuron-essential genes were also identified in our previous screen in iPSC-derived neurons^1^; others were unique to the present *in vivo* screen. To determine if these hits are reproducible at different time points and are CRISPRi-dependent, we repeated the screen in both CRISPRi mice and non-transgenic wild-type mice and determined hits at different time points after injection. Most of the top hits were replicated across independent CRISPRi mice, whereas none were hits in wild-type mice, confirming high reproducibility of the top hits and CRISPRi dependency (Fig. 4c, Supplementary Table 2). Thus, the introduction of selective amplification of sgRNAs from Cre-expressing cells vastly improved the performance of the screen relative to our previous strategy (Fig. 1d).

### Validation of *Hspa5* as a neuron-essential gene in mice

**Fig. 5:**
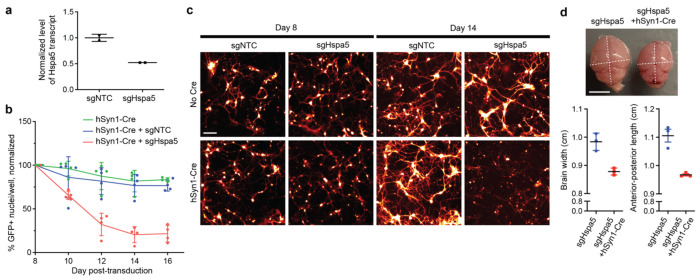
Validation of *Hspa5* as an essential gene in mouse neurons **a,** Cultured mouse embryonic fibroblasts (MEFs) from constitutive dCas9-KRAB mice were transduced with lentivirus (pMK1334) containing an sgRNA targeting *Hspa5* (sgHspa5) versus a non-targeting control sgRNA (sgNTC). *Hspa5* mRNA levels were assayed by qPCR and normalized to the sgNTC control (mean ± s.d., n=2 technical replicates). **b**, Survival of primary neurons cultured from conditional CRISPRi mice following transduction with AAV-hSyn1-Cre alongside AAV-pAP215 containing sgHspa5 versus sgNTC (mean ± s.d., n = 4 wells). **c**, Representative images of primary neurons at 8 or 14 days post transduction with the AAVs as in panel (b). Cells were co-transduced with constitutively expressed cytosolic mScarlet, which reveals fine neuronal processes, displayed in this panel. Scale bar 125 μm. **d,** Representative brains of conditional CRISPRi mice 16 days after neonatal ICV injection of AAV-pAP215-sgHspa5 with or without hSyn1-Cre, and brain measurements (mean ± s.d., n = 3 independent mice). Scale bar, 5 mm. Gross motor phenotypes are shown in Supplementary Videos 1 and 2.

We selected *Hspa5*, a top hit that was not previously identified as a hit in iPSC-derived neurons, for individual validation. In mouse embryonic fibroblasts expressing CRISPRi machinery, we confirmed that an sgRNA targeting *Hspa5* (sgHspa5) suppresses expression of the endogenous *Hspa5* transcript (Fig. 5a). In primary neurons cultured from conditional CRISPRi mice, AAVs delivering sgHspa5 led to marked Cre-dependent neuronal death within 2 weeks of expression (Fig. 5b,c). Furthermore, injection of this sgRNA into neonatal mice led to a severe motor phenotype after approximately 2 weeks in mice co-expressing hSyn1-Cre, but not the sgRNA alone (Supplementary Videos 1 and 2), and the brains from mice with sgHspa5 + hSyn1-Cre were markedly smaller in size relative to sgHspa5-only littermates (Fig. 5d). This confirms the capability of our platform to uncover neuron-essential genes.

## Discussion

In summary, we have established an *in vivo* platform for cell-type selective CRISPR-based screening *in vivo* in mouse tissues with excellent scalability due to the amplification of sgRNA sequences from AAV episomes isolated from tissues of interest. Our pilot screen of >2,000 genes to uncover neuron-essential genes in the brain found high reproducibility of top hits between individual mice and uncovered neuron-essential genes not previously identified in iPSC-derived neurons.

While we identified gene perturbations that reduced neuronal survival, future applications in the context of mouse models of neurodegenerative diseases involving neuronal loss have the potential to uncover genetic perturbations rescuing neuronal survival, and thus point to novel therapeutic strategies. To model therapeutic intervention at different stages of disease progression, temporal control of gene perturbation can be achieved by delivering sgRNA libraries at later time points via intravenous delivery (for example through retro-orbital injection), or by crossing lox-Stop-lox CRISPRi/a mice to Cre-ERT2 driver mice, which will enable cell type-specific activation of genetic perturbation by tamoxifen administration in mice where sgRNA libraries were introduced at birth.

A limitation of the delivery of sgRNAs by non-integrating AAVs is that AAV episomes will be lost progressively during cell division. When targeting proliferating cell types, this will reduce the fraction of cells from which phenotypic information is collected and limited the scalability of the screen. This is a minor concern in the context of the brain, where most cell types are not strongly proliferative. One strategy to overcome this limitation in applications to other tissues is the incorporation of a transposase system to integrate AAVs into the host genome, as demonstrated previously^15^.

An area for future optimization is the multiplicity of infection for sgRNA libraries. CRISPR-based screens in cultured cells are typically carried out at a low multiplicity of infection to ensure that most cells receive only one sgRNA. In contrast, AAV in the CNS targets heterogenous cell populations across a complex tissue architecture with stark differences in tropism, which makes controlling multiplicity of infection challenging across large brain regions; a low multiplicity of infection for a one brain region of high viral tropism may result in effectively no transduction of some other cell types. Our present screen was carried out under typical conditions of moderate to high multiplicity of infection in most brain regions. We hypothesize that this did not thwart the identification of hit genes, since the majority of sgRNA library elements can be expected not to cause a phenotype, and because the vast number of infected neurons ensured that each sgRNA was phenotypically evaluated in a large number of cells, each time randomly paired with different sgRNAs, such that phenotypes of individual sgRNAs were still readily discernible. Future *in vivo* screening strategies in the CNS must balance the size of the library, the target population(s), and the phenotypes of interest in deciding on a multiplicity of infection.

In future applications, our approach can be used to interrogate different cell populations in the brain, taking advantage of the growing repertoire of AAV capsids^16^ and cell type-specific promoters to target subtypes of neurons or glial cells. While our first screen was based on survival as a phenotype, *in vivo* screening can be combined with fluorescence-activated cell sorting (based on fluorescent reporters, immune-staining for endogenous factors, or the use of indicator dyes) or single-cell RNA sequencing to interrogate a broad range of complex cellular phenotypes in the physiological context.

## Methods

### Animals

All mice were maintained according to the National Institutes of Health guidelines and all procedures used in this study were approved by the UCSF Institutional Animal Care and Use Committee. Mice were housed on a 12-h light/dark cycle at 22-25 °C, 50-60% humidity, and had food and water provided *ad libitum*. Mice were randomly assigned for the experimental groups at time of injection and both male and female mice were used. In accordance with approved protocol, mice were monitored post injection and if signs of distress appeared, mice were documented and euthanized promptly. The mice used in this study are LSL-dCas9-KRAB mice (B6;129S6-*Gt(ROSA)26Sor*^tm2(CAG-cas9^*^/ZNF10^*^)Gers^/J, RRID: IMSR_JAX:033066)^14^, dCas9-KRAB mice (B6.Cg-*Igs2*^tm1(CAG-mCherry,-cas9/ZNF10^*^)Mtm^/J, RRID: IMSR_JAX:030000), and B6 wild-type mice (C57BL/6J, RRID: IMSR_JAX:000664). The mice tested by lentiviral delivery were neonates from a cross between B6(SJL)-*Apoe*^tm1.1(APOE^*^4)Adiuj^/J (RRID:IMSR_JAX:027894) and *App*^NL-G-F^ knock-in mice^19^.

### Plasmids

The screening vector pAP215, diagrammed in Figure 1b, was generated using the pAAV-U6-sgRNA-CMV-GFP plasmid as the starting backbone (a gift from Hetian Lei, Addgene plasmid # 85451, RRID:Addgene_85451)^20^. We replaced the hU6-sgRNA cassette with the mU6-sgRNA cassette from pMK1334 (ref. ^1^) (Addgene plasmid #127965, RRID:Addgene_127965) by restriction cloning between sites MluI and XbaI. The mU6 was then replaced using Gibson Assembly with a gene block (gBlock, IDT technologies) containing a modified mU6 which combined the first 303 nucleotides from a separate mU6 sequence derived from pMU6-gRNA (gatccgacgccgccatctctaggcccgcgccggccccctcgcacagacttgtgggagaagctcggctactcccctgccccggttaatttgcatataatatttcctagtaactatagaggcttaatgtgcgataaaagacagataatctgttctttttaatactagctacattttacatgataggcttggatttctataagagatacaaatactaaattattattttaaaaaacagcacaaaaggaaactcaccctaactgtaaagtaattgtgtgttttgagactataaatatcccttggaga, sequence synthesized by referencing Addgene plasmid # 53187 (ref. ^21^) with a BstXI restriction site (ccaccttgttg). The CMV-EGFP module was replaced by EF1a-2xmycNLS-tagBFP2 from pMK1334 by Gibson Assembly. The W3 terminator was cloned from Cbh_v5 AAV-saCBE C-terminal (a gift from David Liu, Addgene plasmid #137183, RRID:Addgene_137183)^22^ and inserted at the EcoRI-XhoI multiple cloning site by Gibson assembly. The hGH was replaced by the SV40 from pMK1334. The Lox66 and Lox71 sequences and their orientation was copied from the pFrt-invCAG-Luc (a gift from Ivo Huijbers, Addgene plasmid #63577, RRID:Addgene_63577)^23^ and were inserted along with the 175-bp intervening spacer as a gBlock at the xbaI-KpnI multiple cloning site.

### sgRNA cloning

To generate a functional mouse sgRNA pooled library of the pAP210 and pAP215 plasmids, we transferred the sgRNA sequences from the mCRISPRi-v2 M1 (Kinases, Phosphatases, and Drug Targets) gRNA pooled library (Addgene pooled library #83989)^10^. 20 μg of the library was digested with BstXI (Thermo Scientific, ER1021) and Bpu1102I (Thermo Scientific, ER0091). The guide-encoding inserts (84 bp) were resolved on a 4-20% Novex TBE gel (Invitrogen, EC62252BOX) and precipitated with GlycoBlue and sodium acetate. Inserts were washed with ethanol after precipitation and then eluted in DNase- and RNase-free water. The backbone vector, pAP215, was digested in parallel with BstXI and Bpu1102I, resolved on a 1% agarose gel, and purified from the gel (Zymo Research, D4001). The vectors and insert guides were annealed for 16 hrs overnight using T4 ligase (New England Biolabs, M0202L) at a 1:2 molar ratio of vector to insert, and then purified with sodium acetate and ethanol washing. After the final wash, the library product was transformed into chemically competent *E. coli* (Takara, 636763) and 10 colonies were picked at random to ensure that each colony was unique. Upon confirmation, the library product was electroporated into Mega-X competent cells (Invitrogen, C640003) and grown overnight, and a portion of the culture was plated to determine if a coverage of at least 250 colonies per guide was achieved, followed by growth of the remainder of the culture in 1 L of LB for 16 hrs, and purification of the library using ZymoPURE II Plasmid Gigaprep Kit (Zymo Research, D4204).

Individual sgRNAs were cloned in the pAP215 backbone digested with BstXI and Bpu1102I using annealed oligos. The protospacer sequences for the specific sgRNAs used in this study include: sgCreb1 (5’-GGCTGCGGCTCCTCAGTCGG-3’), sgHspa5 (5’-GAACACTGACCTGGACACTT 3’), and a non-targeting control (sgNTC) (5’-GGATGCATAGATGAACGGATG-3’).

### AAV packaging, purification, and titering

The pAP215-M1 library was packaged into AAV for transduction into neonatal mice as follows. Two 15-cm dishes were each seeded with 1.5×10^7^ HEK 293T cells (ATCC, CRL-3216) in 25 ml DMEM complete medium: DMEM (Gibco, 11965-092) supplemented with 10% FBS (VWR, 89510, lot: 184B19), 1% penicillin-streptomycin (Gibco, 15140122), and 1% GlutaMAX (Gibco, 35050061). The next day, 20 μg of pAdDeltaF6 (a gift from James M. Wilson, Addgene plasmid # 112867, RRID:Addgene_112867), 7 μg of pAP215-M1 library plasmid, 7 μg of pUCmini-iCAP-PHP.eB (a gift from Viviana Gradinaru, Addgene plasmid #103005, RRID:Addgene_103005)^24^, and 75 μl of 1 mg/ml polyethenylamine (PEI) (Linear, MW 25,000, Polysciences, 23966) were diluted into 4 ml of Opti-MEM (Gibco, 31985062), gently mixed, and incubated at room temperature for 10 min. The PEI/DNA transfection complex was then pipetted drop-wise onto the HEK 293T cells. After 24 hours, the media was replaced with 27 ml of fresh Opti-MEM.

AAV precipitation was performed as previously described^25^, with modifications. Cold 5× AAV precipitation solution (40% polyethylene glycol (Sigma-Aldrich, 89510) and 2.5 M NaCl) was prepared. The cells and media were triturated and collected (~30 ml) into a 50 ml conical tube, followed by addition of 3 ml chloroform and vortexing for approximately 30 seconds. The homogenate was centrifuged at 3,000g for 5 min at room temperature, and the aqueous (top) phase was transferred to a new 50 ml conical tube and 5× AAV precipitation solution was added to a final 1× concentration, followed by incubation on ice for at least 1 hour. The solution was centrifuged at 3,000g for 30 min at 4°C. The supernatant was completely removed and the viral pellet was resuspended in 1 ml of 50 mM HEPES and 3 mM MgCl_2_, and incubated with 1 μl DNase I (New England Biolabs, M0303S) and 10 μl RNase A (Thermo Scientific, EN0531) at 37°C for 15 min. An equal volume of chloroform was added, followed by vortexing for 15 sec, and centrifuged at 16,000g for 5 min at RT. Using 400 μl at a time, the aqueous phase was passed through a 0.5-ml Amicon Ultra Centrifugal Filter with a 100 kDa cutoff (Millipore, UFC510024) by 3 min of centrifugation at 14,000g, followed by buffer exchange twice with 1× DPBS. This preparation yields 40 μl of AAV at a titer of approximately 2×10^12^ viral genomes per μl Titering was performed by quantitative RT-PCR as previously described^26^, using primers listed in Supplementary Table 4.

To prepare AAV for testing in primary neuronal cultures, HEK293T cells were seeded into a 6-well format containing 1.5 ml of DMEM complete media. The cells were transfected with 1 μg pAdDeltaF6, 350 ng pUCmini-iCAP-PHP.eB, and 350 ng of AAV transgene as above. Approximately 48 hours after transfection, the cells and media were collected in 2 ml microfuge tube, 200 μl of chloroform was added to each tube, vortexed for 15 sec, and centrifuged at 16,000g for 5 min at room temperature. The aqueous (top) phase was transferred to a new tube and AAV precipitation solution was added to 1× dilution, and incubated on ice for at least one hour. The precipitated AAV was centrifuged at 16,000g for 15 min at 4°C, the supernatant was removed, the pellet was resuspended in 100 μl of 1× PBS, and centrifuged again at 16,000g for 1 min to remove excess debris, and the supernatant (purified virus) was transferred to a new microfuge tube. 10 μl purified virus was used per well in primary neuronal cultures in a 24-well format.

### Intracerebroventricular injection

Intracerebroventricular (ICV) injections were performed as previously described, with minor modifications^27^. Briefly, neonate mice were placed on a gauze-covered frozen cold pack and monitored for complete cryoanesthesia. The scalp was gently cleaned with an alcohol swab. 1 μl of each AAV with 0.1% trypan blue was loaded into 10 μl syringe (Hamilton, 1701) into a final volume of 2 μl for injection. The syringe was equipped with a 33-gauge beveled needle (Hamilton, 7803-05, 0.5 inches in length). The needle was inserted through the skull 2/5 of distance of the lambda suture to the eye and to a depth of 3 mm to target the left lateral ventricle. Following a one-time unilateral injection, the neonate was placed on a warming pad for recovery and returned to the parent cage.

### Retroorbital injection

3 μl of purified AAV sgRNA library was diluted into 100 microliters of 1x PBS and loaded into a 29G x 0.5 inches 1cc insulin syringe. Mice were briefly anesthetized into a drop chamber containing gauze soaked in 0.5 ml isoflurane, followed by intravenous injection of the 100 μl of diluted AAV into the retroorbital space accessed medial to the mouse right globe. The mice were monitored for awakening returned to their cage for recovery. All eight mice injected were 9-week-old males.

### sgRNA recovery, sequencing, and analysis

Animals were euthanized using CO_2_, and their whole brains were removed and stored at −80°C. The sex of the mice was recorded prior to euthanasia. Each brain was placed in a PYREX 7 ml Dounce Homogenizer (Corning, 7722-7) with 2 ml of TRIzol (Invitrogen, 15596026) and thoroughly homogenized using the A pestle (0.0045 nominal clearance) for 10 or more strokes. The homogenate was divided into two 1.5 ml centrifuge tubes and 0.2 ml of chloroform was added to each tube, followed by centrifugation at 12,000g for 15 min at 4°C. The aqueous phase was transferred to a new tube, and 0.5 ml of isopropanol was added and centrifuged at 12,000g for 10 min. The supernatant was discarded and the pellet was resuspended in 1 ml of 75% ethanol in dNase/RNase-free water, then vortexed briefly, and then spun down at 7,500g for 5 min. The supernatant was then removed and the pellet was allowed to air dry for 10 mins, and then resuspended in 100 μl of dNase/RNase-free water and incubated with 1 μl of RNase A (Thermo Scientific, EN0531) at 37°C overnight. The sample was then column purified by Zymo DNA Clean & Concentrator-25 kit (Zymo Research, D4033) and eluted in 50 μl of dNase/RNase-free water to yield recovered viral DNA.

The purified episomal DNA and the starting plasmid pooled library were PCR amplified with adapter sequences (Supplementary Table 4), sequenced on the Illumina HiSeq4000 using a custom sequencing primer (oMK734_HS4Kmirror_CRISPR_SP:CCACTTTTTCAAGTTGATAACGGACTAGCCTTATTTAAACTTGCTATGCTGT) at the UCSF Center for Advanced Technologies, and analyzed as previously described^1^. Phenotypes and adjusted *p*-values for each gene were generated using our previously established analysis pipeline (https://kampmannlab.ucsf.edu/mageck-inc)^1^. The PCR was performed using Q5 High-Fidelity 2X Master Mix (NEB, M0492L) with conditions described in Supplementary Table 4.

### Lentivirus packaging, purification, and injection

The pLG15 vector containing a non-targeting control sgRNA was packaged into lentivirus as previously performed^17^ by transfecting 10 μg of the transfer plasmid and 10 μg of lentiviral packaging plasmids (containing 1:1:1 pRSV, pMDL, pVSVG) into 1.0×10^7^ HEK293T cells cultured in a 10-cm dish in DMEM complete medium. 48 hours after transfection, the virus was precipitated from the media supernatant using Lentivirus Precipitation Solution (Alstem, VC100) and resuspended in 50 μl of PBS. For ICV injection, 4 μl of virus was used for each neonatal mouse. Mouse brains were extracted on day 14 and sectioned coronally.

### Mouse cortical neuron primary cultures and immunocytochemistry

Neonates were briefly sanitized with 70% EtOH and decapitated using sharp scissors, and the brains were removed and placed into cold HBSS (Gibco, 14175095). The meninges were removed under a dissecting microscope, and the cortices were transferred to a 15-ml conical tube containing 10 ml of 0.25% Trypsin-EDTA (Gibco, 25200056) and incubated at 37°C for 30 min. The trypsin was removed and the brains were gently rinsed twice in 5 ml of DMEM complete media, followed by trituration of brains in 5 ml of DMEM complete media using a glass Pasteur pipette (VWR, 14672-380). The triturated tissue was resuspended of DMEM complete media and filtered through a 40 μm nylon cell strainer (Corning, 352340). Approximately one brain was plated across each BioCoat Poly-D-Lysine 24-well TC-treated plate (Corning, 356414). The following day, day in vitro 1 (DIV1), the DMEM complete media was replaced with neuronal growth media composed of Neurobasal-A Medium (Gibco, 10888022), 1× B-27 Supplement minus vitamin A (Gibco, 12587010), GlutaMAX Supplement (Gibco, 35050079), and 1% penicillin-streptomycin (Gibco, 15140122). On DIV2, the cultures were further supplemented with cytarabine to a final concentration of 1 mM (Thermo Scientific Chemicals, 449561000). The primary neuronal cultures were transduced with AAV on DIV4 and imaged starting 4 days after transduction, every other day until day 16 post-transduction (DIV20).

### Mouse embryonic fibroblast generation and lentiviral transduction

Mouse embryonic fibroblasts (MEFs) were harvested at approximately embryonic day 15 from a pregnant mouse containing homozygous constitutive dCas9-KRAB machinery. The heads and internal organs were removed and the embryos were dissected with sharp scissors into pieces ranging from 2-3 mm. The tissues were incubated with 10 ml 0.25% Trypsin-EDTA at 37°C for 30 mins. The trypsin was then carefully aspirated and replaced with 10 ml DMEM complete media, followed by vigorous pipetting, and filtering through a 40 μm nylon cell strainer (Falcon, 352340). This cell suspension was then centrifuged at 200g for 5 mins, and resuspended in 10 ml DMEM complete media, and plated on a 10 cm tissue culture petri dish (Corning, 353003).

The sgRNA sequence targeting *Hspa5* was inserted into the pMK1334 lentiviral backbone^1^ using the strategy described in the “sgRNA cloning” section above. 1 mg of plasmid was packaged into lentivirus using 1x10^6^ HEK293T cells plated in a 35 mm well, precipitated after 48 hours, and resuspended in 200 μL 1x PBS. 50 μL of the virus was used to transduce MEFs plated in a 12-well format, and the cells were selected with 2 μg/ml puromycin (Sigma-Aldrich, P9620) and harvested 5 days after transduction. The cells were then collected for RNA isolation and quantitative RT-PCR.

### RNA isolation and quantitative RT-PCR

RNA was isolated with the Zymo Quick-RNA Microprep Kit (Zymo Research, R1050). Samples were prepared for qPCR in technical duplicates in 10-μl reaction volumes using SensiFAST SYBR Lo-ROX 2× Master Mix (Bioline, BIO-94005), custom qPCR primers from Integrated DNA Technologies used at a final concentration of 0.2 μM and cDNA diluted at 1:20 by volume. qPCR was performed on a Bio-Rad CFX96 Real Time System C1000 Touch Thermocycler. The following cycles were run (1) 98°C for 3 min; (2) 95°C for 15 s (denaturation); (3) 60°C for 20 s (annealing/extension); (4) repeat steps 2 and 3 for a total of 39 cycles; (5) 95°C for 1 s; (6) ramp 1.92°C s^−1^ from 60°C to 95°C to establish melting curve. Expression fold changes were calculated using the ΔΔCt method, normalizing to housekeeping gene *Gapdh*. RT-qPCR primers are listed in Supplementary Table 4.

### Mouse brain immunohistochemistry

Whole brains were removed and fixed overnight at 4°C in 4% paraformaldehyde (Electron Microscopy Sciences, 15710) diluted in 1× PBS. The following day, the fixative was replaced with 30% sucrose dissolved in 1× PBS for at least 48 hours. Fixed brains were blotting dry, cut down the midline with a razorblade, and mounting into a cryomold (Epredia, 2219) using OCT compound (Sakura Finetek, 4583). To snap freeze, cryomolds were partially submerged in a pool of 2-propanol cooled by a bed of dry ice. Brains were sectioned in the sagittal plane at 40 μm on a cryostat (Leica, CM1950) with a 34° MX35 Premier+ blade (Epredia, 3052835). The resulting brain sections were stored free-floating in 1× PBS + 0.05% NaN_3_ at 4°C. When ready for staining, representative brain sections were wasted three times in 1× PBS and incubated in a 24 well plate at room temperature for one hour in blocking buffer: 10% goat serum (Gibco, 16210064), 1% BSA (Sigma-Aldrich, A7906), and 0.3% Triton X-100 (Sigma-Aldrich, T8787) diluted in 1× PBS. The brain sections were incubated in primary antibodies diluted in blocking buffer overnight at 4°C on a gentle shaker. The sections were washed three times in 1× PBS, then incubated in secondary antibodies for 2 hours at room temperature in the dark on a gentle shaker. Sections were washed three times in 1× PBS and moved to charged glass microscope slides (Fisher Scientific, 1255015). After PBS was removed, Fluoromount-G with DAPI mountant (Invitrogen, 00-4959-52) was added, and a No. 1.5 coverslip (Globe Scientific, 1415-15) was applied. Slides were dried at room temperature in the dark overnight and sealed with nail polish. For experiments without DAPI, ProLong Gold Antifade mountant (Invitrogen, P10144) was used instead. For experiments with Hoechst instead of DAPI, sections were lastly incubated for 15 mins in Hoechst 33342 (BD Pharmingen, 561908) diluted 2 μg/ml in 1× PBS, then washed 3 times in 1× PBS before mounting using ProLong Gold mountant.

The following primary antibodies were used: rabbit anti-CREB (1:1,000 dilution, clone: 48H2, Cell Signaling Technologies, 9197), rabbit anti-SOX9 (1:2,000 dilution, polyclonal, EMD Millipore, AB5535), rabbit anti-Cre recombinase (1:500 dilution, clone: D7L7L, Cell Signaling Technologies, 15036), guinea pig anti-NeuN (1:500 dilution, polyclonal, Synaptic Systems, 266004), alpaca FluoTag-Q anti-TagFP nanobody (reacts to mTagBFP2 but not eGFP, 1:500 dilution, clone: 1H7, Alexa647 pre-conjugated, NanoTag Biotechnologies, N0501-AF647-L). A different antibody for mTagBFP2 was used in Extended Data Fig. 2: rabbit anti-tRFP (1:1,000 dilution, polyclonal, Evrogen, AB233). The following secondary antibodies were used: goat anti-rabbit IgG Alexa Fluor 488 (1:1,000 dilution, Invitrogen A-11034), goat anti-rabbit IgG Alexa Fluor 568 (1:1,000 dilution, Invitrogen, A-11011), goat anti-rabbit IgG Alexa Fluor 647 (1:1,000 dilution, Invitrogen, A-21245), goat anti-guinea pig IgG Alexa Fluor 488 (1:1,000 dilution, Invitrogen, A-11073), goat anti-guinea pig IgG Alexa Fluor 647 (1:1,000 dilution, Invitrogen, A-21450). All secondary antibodies were highly cross-absorbed.

### Immunocytochemistry

Cells were fixed in 4% paraformaldehyde for 10 min, washed briefly with 1x PBS, and incubated in blocking buffer (5% normal goat serum + 0.1% Triton X-100) for 15 min, all at room temp. Rabbit anti-CREB was applied at 1:2,000 dilution and incubated overnight at 4°C. The cells were gently rinsed five times with 1x PBS, incubated for 1 hour with goat anti-rabbit IgG Alexa Fluor 488, rinsed five times with 1x PBS, and imaged.

### Microscopy, image segmentation, and analysis

Slides containing brain sections were imaged using a Zeiss AxioScan.Z1 with a Zeiss Colibri 7 unit, ×20/0.8 NA objective lens, 5-30 ms exposure, 1×1 binning and 25-100% intensity using 425-nm, 495-nm, 570-nm and 655-nm lasers, running ZEN version 2.6 software. The images were imported into QuPath (version 0.4.2) for analysis. To identify overlap between BFP, NeuN, and Sox9, a representative region of the cortex was outlined and the nuclei were segmented on the DAPI channel using the ‘Cell detection’ module without expansion of the nuclei to develop virtual cell boundaries. Classifiers were created to distinguish BFP-, NeuN-, and Sox9-positive cells, and applied sequentially. Cells containing overlapping NeuN and Sox9 were considered to be neurons and only cells exclusively containing Sox9 were considered astrocytes.

To measure Creb1 levels, a 0.75 mm diameter circular region of the frontal cortex of each brain was selected, and the nuclei were segmented on the DAPI channel as above. The measurements for the segmented nuclei were exported. The mean fluorescence intensity for the anti-Creb1 channel was obtained selected by the top 200 nuclei of highest anti-mTagBFP2 fluorescence intensity.

For mouse primary neurons transduced with AAV, live imaging was performed every other day using an ImageXpress Micro Confocal HT.ai High-Content Imaging System (Molecular Devices). The imaging chamber was warmed to 37°C and equilibrated with 5% CO_2_. The system used an Andor Zyla 4.5 camera with a Plan Apo ×10/0.45NA objective lens, an 89 North LDI laser illumination unit, 10-500 ms exposure time, 1×1 binning, and 10% laser intensity using 405-nm, 475-nm, and 555-nm lasers, running MetaXpress (version 6.7.1.157). Resulting images were imported into Cell Profiler (version 4.2.1)^28^ and analyzed using a custom pipeline. hSyn1-Cre-GFP+ nuclei were segmented using the ‘IdentifyPrimaryObjects’ module, with expected diameter 8-40 pixels, using an Adaptive threshold (size 50) and the Minimum Cross-Entropy method, with a 1.5 smoothing scale, 1.0 correction factor, and lower- and upper-bound threshold at 0.435 and 1, respectively. Segmented objects were exported, and counted in each field, then summed across all fields within a well to calculate the number of objects per well (n=29 fields per well, n=4 wells per condition), using a custom R script. This was repeated for each timepoint. Data was normalized to fluorescent intensity at day 8 (as before that day, fluorescence intensity increased linearly with time in all channels as cells manufactured fluorescent proteins) and percentage change was calculated for each well from day 8, for subsequent timepoints through day 16.

### Statistics and Reproducibility

No statistical methods were used to pre-determine sample sizes, but our sample sizes are similar to those reported in previous publications, as cited in the main text. Numbers of replicates are listed in each figure. No repeat measurements were made on the same samples. Data were assumed to be normally distributed except for instances within the MAGeCK-iNC pipeline^29^ where the two-sided Mann-Whitney U-test (no distributional assumptions) was used to determine statistical significance. The MAGeCK-iNC pipeline was used with FDR < 0.1 and controls for multiple comparisons. For cell culture experiments, randomization was not performed because treatment groups of cells were derived from the same parent population of cells. Data collection and analysis were not performed blinded to the conditions of the experiments. No animals or data points were excluded from the relevant analyses, with the exception of some 4-wk and 12-wk timepoint screens which were excluded due to poor technical quality (n= 7, of 30 total). Poor technical quality was defined as fewer than 1 million reads and/or had a poor correlation between the input (AAV) and output (brain) sgRNA counts (R^2 < 0.9). Major findings were validated using independent samples and orthogonal approaches.

## Extended Data

## Supplementary Material

Supplement 1

Supplement 2

Supplement 3

Supplement 4

Supplement 5

Supplement 6

7

## Data Availability

All Data are available from the corresponding author (MK), and will be made publicly available at the UCSF Dryad data repository upon publication. There are no restrictions on data availability.
