# Supplementary material for: CRISPR screening by AAV episome-sequencing (CrAAVe-seq) is a highly scalable cell type-specific in vivo screening platform
Source: bioRxiv. 2024 Dec 18:2023.06.13.544831. Preprint. [Version 4] doi: 10.1101/2023.06.13.544831 (PMC10312723; doi:10.1101/2023.06.13.544831)
Supplement: Supplement 9 [file NIHPP2023.06.13.544831v4-supplement-9.pdf]

bioRxiv preprint doi: https://doi.org/10.1101/2023.06.13.544831; this version posted December 19, 2024. The copyright holder for this preprint (which was not certified by peer review) is the author/funder, who has granted bioRxiv a license to display the preprint in perpetuity. It is made available under aCC-BY 4.0 International license.

Supplementary Materials

Supplementary File 1: Annotated sequence of CrAAVe-seq plasmid pAP215. A map of pAP215 is provided in a GenBank file format.

Supplementary Table 1: Summary of mice injected with AAV across different studies
Lists all mice used in the study, time points, biological sex, and virus amounts. For mice used for CRISPR screens, the number of sequencing reads obtained from each sample is included.

Supplementary Table 2: The CRISPR screen results of cohorts
Provides the results file of the 'crispr_screen' pipeline applied to the different cohorts of mice reported in this manuscript.

Supplementary Table 3: CRISPR screen results for individual mice
Provides the knockdown phenotype (log2fc), pvalue, FDR, and Gene Score, obtained from the output of the 'crispr_screen' pipeline for all individual mice relative to input the AAV library, with each tab representing a different cohort of mice.

Supplementary Table 4: List of primers used for sgRNA amplification, next generation sequencing, and RT-qPCR
Provides the primer sequences for sgRNA amplification, the custom sequencing primers for Illumina sequencing, and the primers used for RT-qPCR of *Hspa5*, *Rabggta*, and *Gapdh*.

Supplemental Table 5: Values generated from bootstrap analyses for CaMKII-Cre and hi56i-Cre cohorts.
Three tabs each for bootstrap analyses of the CaMKII-Cre and hi56i-Cre cohorts reported in Fig. 6 are included. "Recovery by mouse number" tab shows the fraction of hits (frac_overlapping) that overlap with the hits of the full cohort when sampling different numbers of mice (subset). "All bootstraps" shows the total number (num_tests) and fraction (frac_tests) of all bootstraps that recover each hit of the full cohort. "Hits by subsample" shows the number (num_tests) and fraction (frac_tests) of bootstraps that recover each hit of the full cohort when sampling different numbers of mice (subset). Each test was run 50 times (replicate).

Supplementary Video 1: Gross motor phenotypes in sgHspa5 + hSyn1-Cre injected mice
LSL-CRISPRi neonates were co-injected by ICV with AAV containing sgHspa5 (in pAP215) and AAV containing hSyn1-Cre. At 16 days post injection, mice displayed gross motor phenotypes,

bioRxiv preprint doi: https://doi.org/10.1101/2023.06.13.544831; this version posted December 19, 2024. The copyright holder for this preprint (which was not certified by peer review) is the author/funder, who has granted bioRxiv a license to display the preprint in perpetuity. It is made available under aCC-BY 4.0 International license.

as recorded in the video. After video capture, mice were immediately euthanized as per IACUC protocol. Mice were littermates to those shown in Supplementary Video 2 and injected and recorded at the same timepoint.

Supplementary Video 2: Lack of gross motor phenotypes in sgHspa5 only injected mice
LSL-CRISPRi neonates were co-injected by ICV with AAV containing sgHspa5 (in pAP215) and an equivalent volume of phosphate buffered saline. At 16 days post injection, mice displayed normal motor phenotypes, as recorded in the video. Mice were littermates to those shown in Supplementary Video 1 and injected and recorded at the same timepoint.

Supplementary Material References

36. Duan, Y. *et al.* The Clustered, Regularly Interspaced, Short Palindromic Repeats-associated Endonuclease 9 (CRISPR/Cas9)-created MDM2 T309G Mutation Enhances Vitreous-induced Expression of MDM2 and Proliferation and Survival of Cells. *J Biol Chem* **291**, 16339–16347 (2016).
37. Kabadi, A. M., Ousterout, D. G., Hilton, I. B. & Gersbach, C. A. Multiplex CRISPR/Cas9-based genome engineering from a single lentiviral vector. *Nucleic Acids Res* **42**, e147 (2014).
38. Levy, J. M. *et al.* Cytosine and adenine base editing of the brain, liver, retina, heart and skeletal muscle of mice via adeno-associated viruses. *Nat Biomed Eng* **4**, 97–110 (2020).
39. Huijbers, I. J. *et al.* Using the GEMM-ESC strategy to study gene function in mouse models. *Nat Protoc* **10**, 1755–1785 (2015).
40. Biebl, M. M. *et al.* NudC guides client transfer between the Hsp40/70 and Hsp90 chaperone systems. *Mol Cell* **82**, 555-569.e7 (2022).
41. Negrini, M., Wang, G., Heuer, A., Björklund, T. & Davidsson, M. AAV Production Everywhere: A Simple, Fast, and Reliable Protocol for In-house AAV Vector Production Based on Chloroform Extraction. *Current Protocols in Neuroscience* **93**, e103 (2020).
42. Aurnhammer, C. *et al.* Universal real-time PCR for the detection and quantification of adeno-associated virus serotype 2-derived inverted terminal repeat sequences. *Hum Gene Ther Methods* **23**, 18–28 (2012).
43. Kim, J.-Y., Grunke, S. D., Levites, Y., Golde, T. E. & Jankowsky, J. L. Intracerebroventricular Viral Injection of the Neonatal Mouse Brain for Persistent and Widespread Neuronal Transduction. *JoVE (Journal of Visualized Experiments)* e51863 (2014) doi:10.3791/51863.
44. Gilbert, L. A. *et al.* Genome-Scale CRISPR-Mediated Control of Gene Repression and Activation. *Cell* **159**, 647–661 (2014).
45. Bankhead, P. *et al.* QuPath: Open source software for digital pathology image analysis. *Sci Rep* **7**, 16878 (2017).
46. Stirling, D. R. *et al.* CellProfiler 4: improvements in speed, utility and usability. *BMC Bioinformatics* **22**, 433 (2021).
47. Wang, G. *et al.* Mapping a functional cancer genome atlas of tumor suppressors in mouse liver using AAV-CRISPR-mediated direct in vivo screening. *Sci Adv* **4**, eaao5508 (2018).
