# Supplementary material for: CRISPR screening by AAV episome-sequencing (CrAAVe-seq) is a highly scalable cell type-specific in vivo screening platform
Source: bioRxiv. 2024 Oct 27:2023.06.13.544831. Preprint. [Version 3] doi: 10.1101/2023.06.13.544831 (PMC10312723; doi:10.1101/2023.06.13.544831)
Supplement: Supplementary file 1 [file NIHPP2023.06.13.544831v3-supplement-9.pdf]

bioRxiv preprint doi: https://doi.org/10.1101/2023.06.13.544831; this version posted October 27, 2024. The copyright holder for this preprint (which was not certified by peer review) is the author/funder, who has granted bioRxiv a license to display the preprint in perpetuity. It is made available under aCC-BY 4.0 International license.

Supplementary Material References

32. Duan, Y. *et al.* The Clustered, Regularly Interspaced, Short Palindromic Repeats-associated Endonuclease 9 (CRISPR/Cas9)-created MDM2 T309G Mutation Enhances Vitreous-induced Expression of MDM2 and Proliferation and Survival of Cells. *J Biol Chem* **291**, 16339–16347 (2016).
33. Kabadi, A. M., Ousterout, D. G., Hilton, I. B. & Gersbach, C. A. Multiplex CRISPR/Cas9-based genome engineering from a single lentiviral vector. *Nucleic Acids Res* **42**, e147 (2014).

34. Levy, J. M. *et al.* Cytosine and adenine base editing of the brain, liver, retina, heart and skeletal muscle of mice via adeno-associated viruses. *Nat Biomed Eng* **4**, 97–110 (2020).
35. Huijbers, I. J. *et al.* Using the GEMM-ESC strategy to study gene function in mouse models. *Nat Protoc* **10**, 1755–1785 (2015).
36. Biebl, M. M. *et al.* NudC guides client transfer between the Hsp40/70 and Hsp90 chaperone systems. *Mol Cell* **82**, 555-569.e7 (2022).
37. Negrini, M., Wang, G., Heuer, A., Björklund, T. & Davidsson, M. AAV Production Everywhere: A Simple, Fast, and Reliable Protocol for In-house AAV Vector Production Based on Chloroform Extraction. *Current Protocols in Neuroscience* **93**, e103 (2020).
38. Aurnhammer, C. *et al.* Universal real-time PCR for the detection and quantification of adeno-associated virus serotype 2-derived inverted terminal repeat sequences. *Hum Gene Ther Methods* **23**, 18–28 (2012).
39. Kim, J.-Y., Grunke, S. D., Levites, Y., Golde, T. E. & Jankowsky, J. L. Intracerebroventricular Viral Injection of the Neonatal Mouse Brain for Persistent and Widespread Neuronal Transduction. *JoVE (Journal of Visualized Experiments)* e51863 (2014) doi:10.3791/51863.
40. Gilbert, L. A. *et al.* Genome-Scale CRISPR-Mediated Control of Gene Repression and Activation. *Cell* **159**, 647–661 (2014).
41. Bankhead, P. *et al.* QuPath: Open source software for digital pathology image analysis. *Sci Rep* **7**, 16878 (2017).
42. Stirling, D. R. *et al.* CellProfiler 4: improvements in speed, utility and usability. *BMC Bioinformatics* **22**, 433 (2021).
