# Supplementary material for: Scalable, cell type-selective, AAV-based in vivo CRISPR screening in the mouse brain
Source: bioRxiv. 2023 Jun 27:2023.06.13.544831. Preprint. [Version 2] doi: 10.1101/2023.06.13.544831 (PMC10312723; doi:10.1101/2023.06.13.544831)
Supplement: Supplementary file 1 [file NIHPP2023.06.13.544831v2-supplement-7.pdf]

bioRxiv preprint doi: https://doi.org/10.1101/2023.06.13.544831; this version posted June 27, 2023. The copyright holder for this preprint (which was not certified by peer review) is the author/funder, who has granted bioRxiv a license to display the preprint in perpetuity. It is made available under aCC-BY 4.0 International license.

Supplementary Table legends

Supplementary Table 1: Counts correlation data used to generate Fig. 1c
This table lists the input and output counts from a representative mouse injected with M1 library (the 2,269 gene targeting library) cloned into pAP210 and delivered retro-orbitally. Input refers to the sequenced library before injection, and output refers to the sgRNAs collected from the brain. Data is divided both by each sgRNA (first column) and its corresponding gene target (second column).

Supplementary Table 2: MAGeCK-iNC output for all *in vivo* screens
This table contains the MAGeCK-iNC output file for the both the constitutive and Cre-inducible CRISPRi mice and wild-type mice. Including those used to generate the heatmaps in Fig. 1d and Fig. 4c. This includes: epsilon (effect size), adjusted *p*-value, gene scores (product values), and corresponding gene, per sgRNA-targeting gene index.

Supplementary Table 3: MAGeCK-iNC output for volcano plot in Fig. 4b
These tables provide combined epsilon, adjusted *p*-value, and gene scores (product values) per gene index for combined cohorts of in vivo neuron-selective M1 screens at each timepoint. Each cohort consists of age-matched mice at the 12-wk timepoint pooled together bioinformatically prior to analysis by MAGeCK-iNC analysis pipeline[1]. This data was used to generate the volcano plot in Fig. 4b.

Supplementary Table 4: List of primers used for next generation sequencing and RT-qPCR, and PCR cycling parameters
This table shows the primers and the corresponding Illumina indices for generating sgRNA amplicons for next generation sequencing for both pAP210 and pAP215 constructs, along with the custom sequencing primer. Separate tabs also list the PCR cycling parameters for library generation and the primers used for RT-qPCR for *Hspa5*, *Creb1*, *Gapdh*, and ITRs for viral titering.

Supplementary Video legends

Supplementary Video 1: Gross motor phenotypes in sgHspa5 + hSyn1-Cre injected mice
LSL-CRISPRi neonates were co-injected by ICV with AAV containing sgHspa5 (in pAP215) and AAV containing hSyn1-Cre. At 16 days post injection, mice displayed gross motor phenotypes, as recorded in the video. After video capture, mice were immediately euthanized as per IACUC protocol. Mice were littermates to those shown in Supplementary Video 2 and injected and recorded at the same timepoint.

bioRxiv preprint doi: https://doi.org/10.1101/2023.06.13.544831; this version posted June 27, 2023. The copyright holder for this preprint (which was not certified by peer review) is the author/funder, who has granted bioRxiv a license to display the preprint in perpetuity. It is made available under aCC-BY 4.0 International license.

Supplementary Video 2: Lack of gross motor phenotypes in sgHspa5 only injected mice
LSL-dCas9-KRAB neonates were co-injected by ICV with AAV containing sgHspa5 (in pAP215) and an equivalent volume of phosphate buffered saline. At 16 days post injection, mice displayed normal motor phenotypes, as recorded in the video. Mice were littermates to those shown in Supplementary Video 1 and injected and recorded at the same timepoint.

Supplementary Material References

17. Gilbert, L. A. *et al.* Genome-Scale CRISPR-Mediated Control of Gene Repression and Activation. *Cell* **159**, 647–661 (2014).
18. Goertsen, D. *et al.* AAV capsid variants with brain-wide transgene expression and decreased liver targeting after intravenous delivery in mouse and marmoset. *Nat Neurosci* **25**, 106–115 (2022).
19. Saito, T. *et al.* Single App knock-in mouse models of Alzheimer's disease. *Nat Neurosci* **17**, 661–663 (2014).
20. Duan, Y. *et al.* The Clustered, Regularly Interspaced, Short Palindromic Repeats-associated Endonuclease 9 (CRISPR/Cas9)-created MDM2 T309G Mutation Enhances Vitreous-induced Expression of MDM2 and Proliferation and Survival of Cells. *J Biol Chem* **291**, 16339–16347 (2016).
21. Kabadi, A. M., Ousterout, D. G., Hilton, I. B. & Gersbach, C. A. Multiplex CRISPR/Cas9-based genome engineering from a single lentiviral vector. *Nucleic Acids Res* **42**, e147 (2014).
22. Levy, J. M. *et al.* Cytosine and adenine base editing of the brain, liver, retina, heart and skeletal muscle of mice via adeno-associated viruses. *Nat Biomed Eng* **4**, 97–110 (2020).
23. Huijbers, I. J. *et al.* Using the GEMM-ESC strategy to study gene function in mouse models. *Nat Protoc* **10**, 1755–1785 (2015).
24. Chan, K. Y. *et al.* Engineered AAVs for efficient noninvasive gene delivery to the central and peripheral nervous systems. *Nat Neurosci* **20**, 1172–1179 (2017).
25. Negrini, M., Wang, G., Heuer, A., Björklund, T. & Davidsson, M. AAV Production Everywhere: A Simple, Fast, and Reliable Protocol for In-house AAV Vector Production Based on Chloroform Extraction. *Current Protocols in Neuroscience* **93**, e103 (2020).
26. Aurnhammer, C. *et al.* Universal real-time PCR for the detection and quantification of adeno-associated virus serotype 2-derived inverted terminal repeat sequences. *Hum Gene Ther Methods* **23**, 18–28 (2012).
27. Kim, J.-Y., Grunke, S. D., Levites, Y., Golde, T. E. & Jankowsky, J. L. Intracerebroventricular Viral Injection of the Neonatal Mouse Brain for Persistent and Widespread Neuronal Transduction. *JoVE (Journal of Visualized Experiments)* e51863 (2014).
28. Stirling, D. R. *et al.* CellProfiler 4: improvements in speed, utility and usability. *BMC Bioinformatics* **22**, 433 (2021).
29. Li, W. *et al.* MAGeCK enables robust identification of essential genes from genome-scale CRISPR/Cas9 knockout screens. *Genome Biol* **15**, 554 (2014).
